# Synergistic treatment of TS

**DOI:** 10.18632/oncotarget.20024

**Published:** 2017-08-08

**Authors:** Aditi U. Gurkar, Toren Finkel

**Affiliations:** Aditi U. Gurkar: Department of Medicine, Aging Institute, Division of Geriatric Medicine, Vascular Medicine Institute, University of Pittsburgh, Pittsburgh, PA, USA

**Keywords:** tuberous sclerosis, mTORC1, miR-21, rapamycin, mitochondrial-adaptation

Tuberous Sclerosis (TS) is a rare genetic disorder that affects about 25,000-40,000 individuals in the United States [1]. The disease is characterized by the growth of benign tumors in vital organs such as brain, kidney, heart and skin [1]. Although histologically benign, the tumors are not insignificant, as they can induce a host of secondary complications. For instance, TS patients develop angiomyolipomas, benign lesions on the kidney, that can pose a risk for hemorrhage and can ultimately impair renal function.

TS is caused by point mutations, deletions and insertions in either one of two genes, *Tsc1* or *Tsc2*. TSC1 stabilizes TSC2, forming a protein complex that integrates various nutrient signals and growth inputs. The TSC1-TSC2 complex consolidates these diverse inputs and functions as an inhibitor of the mechanistic target of rapamycin (mTOR), an evolutionarily conserved serine-threonine kinase [2]. mTOR is a known master regulator of metabolism and controls cell growth and survival in response to various signaling inputs. mTOR forms two functionally distinct multi-protein complexes, mTOR complex 1 (mTORC1) that contains the protein, raptor and mTOR complex 2 (mTORC2) that contains the protein, rictor. In TS patients, normal TSC1/TSC2-dependent negative regulation is impaired and mTORC1 is constitutively activated, giving mutant cells a growth advantage over adjacent normal cells [3]. Because of mTORC1 involvement, inhibitors of mTOR such as rapamycin and other rapalogs (rapamycin analogs) have been employed as biological-based therapies to treat TS patients. Although rapamycin therapy does result in a reduction in tumor volume, these effects are not durable, and cessation of treatment often results in tumor recurrence [2]. Therefore, additional, potential combinatorial, therapies are sorely needed for these patients.

Micro-RNA (mi-RNA), a class of non-coding RNA, are predicted to modulate ∼60% of the genes in the human genome. miRNAs are ∼22 nucleotides in length and a single miRNA can regulate the expression of multiple genes thereby affecting a myriad of pathways [4]. Trindade *et. al*. previously tested whether miRNAs are altered in response to mTORC1 inhibition in *TSC*2-deficient cell lines. Using two different platforms to identify miRNAs regulated by rapamycin, they identified six that were dysregulated and classified them as ‘RapamiRs’. This previous study noted that miR-21 was most significantly increased by mTOR-inhibition and was cell type independent [5]. miR-21 has been previously associated with several diseases including cancers. Transcriptional profiling studies have identified that miR-21 controls stress responses, evasion of apoptosis, signal transduction, as well as genes associated with blood vessel morphogenesis and development [6].

In the current study, Lam H.C. *et. al*. tested the hypothesis that miR-21 upregulation might help protect *TSC*2-deficient cells from the cytotoxic effects of mTORC1 inhibition [7]. Interestingly, the authors found that miR-21 is upregulated in TSC2-deficient cells (characterized by high mTORC1 activity) and further induced upon rapamycin treatment (a potent inhibitor of mTORC1). Although *in vitro*, inhibition of miR-21 or treatment with rapamycin reduced proliferation and induced apoptosis, combined miR-21 inhibition and rapamycin therapy appeared to synergistically target TSC2-deficient cells. These results suggest that TSC2-deficient cells treated with rapamycin might have developed a unique reliance on miR-21. The authors then asked what does miR-21 regulate in this unique context? Interestingly, transcriptional profiling hinted that miR-21 was modulating mitochondrial function and potentially allowing the TSC2-deficient cell’s ability to somehow metabolically adapt to rapamycin. Indeed, direct measurements showed that the combination of rapamycin and miR-21 inhibition reduced mitochondrial content, mitochondrial membrane potential, as well as, overall mitochondrial function. More excitingly, this combined treatment significantly reduced *in vivo* growth of xenograft tumors and increased survival in mice by four-fold [7].

One interesting observation by the authors is that in TSC2-deficient cells, miR21 is elevated and yet, further induced by rapamycin. This suggests that this micro-RNA is not regulated by canonical mTORC1 signaling. Previously, others have demonstrated that miR-21 is transcriptionally controlled by AP-1 and STAT3, whereas its maturation is induced by TGF-β and BMP4 [8]. It would be interesting to understand what complex regulatory network controls miR-21 expression after mTORC1 inhibition and what aspect of mitochondrial function is critical for the observed synergistic effects. However, by whatever mechanism(s), this new study from the Henske lab suggests that the path forward for combinatorial therapy of TS patients might have just gotten a bit clearer.
